# Acute Effects of Pimobendan on Cardiac Function in Dogs With Tachycardia Induced Dilated Cardiomyopathy: A Randomized, Placebo-Controlled, Crossover Study

**DOI:** 10.3389/fvets.2021.646437

**Published:** 2021-07-01

**Authors:** Kaitlin Abbott-Johnson, Kursten V. Pierce, Steve Roof, Carlos L. del Rio, Robert Hamlin

**Affiliations:** ^1^QTest Labs, Columbus, OH, United States; ^2^Department of Clinical Sciences, Colorado State University, Fort Collins, CO, United States; ^3^MyoKardia, Brisbane, CA, United States

**Keywords:** pimobendan, dilated cardiomyopathy, mitral regurgitation, inodilator, lusitropy

## Abstract

**Background:** Pimobendan provides a significant survival benefit in dogs with cardiac disease, including degenerative mitral valve disease and dilated cardiomyopathy (DCM). Its positive inotropic effect is well-known, however, it has complex effects and the mechanisms behind the survival benefit are not fully characterized. Secondary hemodynamic effects may decrease mitral regurgitation (MR) in DCM, and the benefits of pimobendan may extend to improved cardiac relaxation and improved atrial function.

**Hypothesis/Objectives:** Our objective was to investigate the acute cardiac effects of pimobendan in dogs with a DCM phenotype. We hypothesized that pimobendan would increase left atrial (LA) contractility, reduce mitral regurgitation, improve diastolic function, and lower circulating NT-ProBNP levels.

**Animals:** Seven purpose-bred Beagles were studied from a research colony with tachycardia induced DCM phenotype.

**Methods:** The effects of pimobendan were studied under a placebo-controlled single-blinded cross-over design. In short, dogs underwent baseline and 3 h post-dose examinations 7 days apart with echocardiography and a blood draw. Dogs were randomized to receive oral placebo or 0.25 mg/kg pimobendan after their baseline exam. Investigators were blinded to treatments until all measurements were compiled.

**Results:** When treated with pimobendan, the dogs had significant increases in systolic function and decreases in MR, compared to when treated with placebo.

There were no detectable differences in left atrial measures, including LA size, LA emptying fraction, LA functional index or mitral A wave velocity. Heart rate decreased significantly with pimobendan compared to placebo. There was also a decrease in isovolumetric relaxation time normalized to heart rate. NT-proBNP levels had a high degree of variability.

**Conclusions:** Improved mitral regurgitation severity and improved lusitropic function may contribute to the reported survival benefit for dogs with cardiac disease administered pimobendan. Pimobendan did not overtly improve LA function as assessed by echocardiography, and NT-proBNP was not significantly changed with a single dose of this medication. Further studies are needed to better characterize LA effects with other imaging modalities, to better quantify the total improvement of MR severity, and to assess chronic use of pimobendan on diastolic function in DCM.

## Introduction

Pimobendan has become an integral tool in the treatment of heart failure in dogs by improving and significantly prolonging their lifespan (1, 2). This phosphodiesterase-3 inhibitor and calcium sensitizer medication has complex effects, and the exact mechanism(s) by which it improves clinical signs and survival remain to be defined. Pimobendan exerts direct effects on both the myocardium and the vasculature, and is described as an “inodilator”—both a positive inotrope and vasodilator (3, 4). While its effects on improving ventricular systolic function are well-known, it has also been suggested that its load modulating properties (e.g., decreased vascular resistance) may be equally or more important in the setting of dilated cardiomyopathy (DCM), characterized by volume/fluid overload (1, 5). Given this pleotropic mechanism, the overarching goal of these experiments was to characterize important but less-described cardiac effects of pimobendan in a model of heart failure.

Left atrial (LA) size and function are widely used as indicators of cardiac disease severity and progression, carrying significant prognostic information. Although LA size appears to decrease with pimobendan administration (6), evidence of a left atrial inotropic effect is sparse. Moreover, echocardiographic evaluation of left atrial pump function can be complicated as LA size is strongly influenced by both ventricular function and loading conditions. For instance, there are reports of increased atrial function under pimobendan treatment, as assessed by LA fractional shortening in two recent feline studies (7, 8), but no evidence of improvement in dogs with degenerative mitral valve disease (DMVD) (9). Therefore, we sought to further investigate the effects of pimobendan on the LA in canine DCM by focusing on multiple echocardiographic indicators of size and function.

While this medication is widely used and considered to be a part of the gold standard treatment for dogs with DMVD ACVIM Stage B2, C, and D, there are conflicting data regarding its effect on mitral regurgitation (MR). A retrospective study of two dogs given pimobendan chronically showed increased regurgitant volume (10) while a study of four dogs with experimentally induced mitral disruption showed decreased regurgitant volume (11), but another study of 19 preclinical DMVD dogs showed no changes in regurgitant volume (12). Other studies show evidence that positive inotropes decrease regurgitant fraction and effective regurgitant orifice area (EROA) in dogs with mitral disruption (13). However, to our knowledge pimobendan's effect on regurgitant volume has not been assessed in DCM.

Older studies have shown that pimobendan can improve isovolumic ventricular relaxation *via* invasive methods using direct left ventricular (LV) pressure measurements (–dP/dt) (14, 15). We sought to further evaluate the lusitropic effects of this medication echocardiographically, using isovolumetric relaxation time (IVRT) and tissue Doppler imaging (TDI).

Pimobendan has been shown to increase aortic flow velocity and fractional shortening while decreasing left ventricular size in healthy dogs with peak effects between 2 and 5 h after oral dosing (16, 17). Therefore, we elected to study the dogs 3 h after dosing to capture the peak pharmacodynamic effects. We hypothesized that pimobendan would decrease LA size and improve function, decrease MR, and have a positive lusitropic effect in dogs with tachypacing induced dilated cardiomyopathy. We also hypothesized that the neurohormonal marker NT-proBNP would change with a single dose of this medication.

## Methods

### Animals

Seven beagle dogs with right ventricular (RV) tachypacing induced cardiac dysfunction were selected to be the subjects of a prospective, single-blinded, randomized placebo-controlled crossover study.

All study activities were approved by QTest Labs' Institutional Animal Care and Use Committee (IACUC). Exclusion/early removal criteria were outlined in study protocols prior to the study. No animals were excluded due to welfare concerns. Seven intact male purpose-bred beagle dogs were enrolled in this study with a weight range of 8.9–13.9 kg (mean 11.1 kg) and age 1–2 years (mean 20 months).

**Figure 1:**
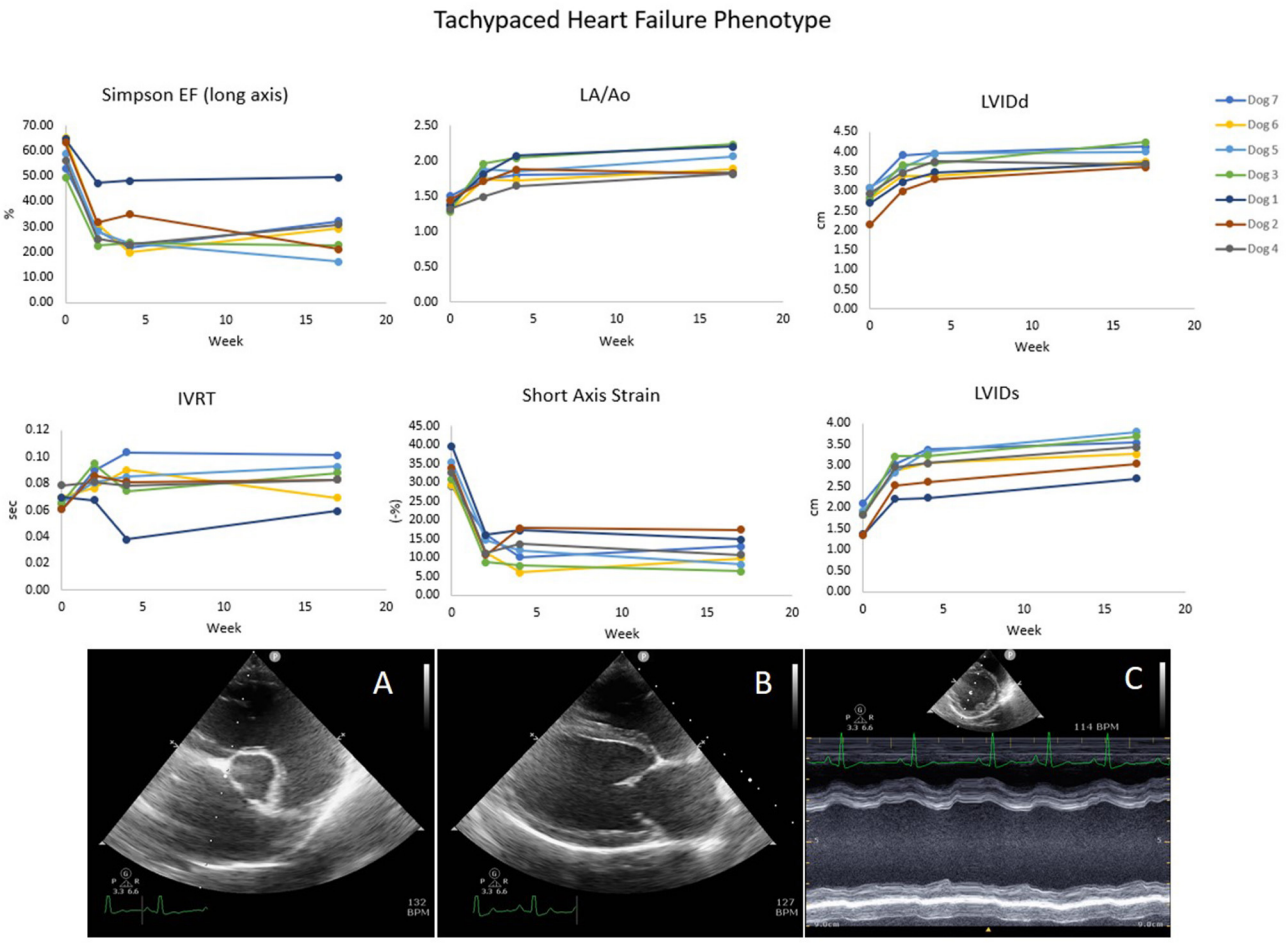
The Tachypaced heart failure phenotype shows decreases in ejection fraction (EF), and strain and increases in IVRT, LA size, and left ventricular diameter. IVRT, isovolumetric relaxation time. **(A)** Right short axis basilar LA:Ao view. **(B)** Right long axis view used for Simpson's EF. **(C)** Right short axis view used for M-mode.

Dogs were implanted with an active-fixation RV apex lead *via* right jugular venous access under fluoroscopic guidance and placement of a pulse generator subcutaneously in a fashion similar to that described in the veterinary literature (18). Dogs were allowed to recover from surgery for at least 3 weeks, and then underwent a well-established tachypacing protocol (RV pacing at 240 pulses per minute (ppm) for 4 weeks, followed by RV pacing at 180 ppm thereafter) in order to induce stable dilated ventricular remodeling, mimicking naturally-occurring DCM (19). All dogs had echocardiograms and NT-proBNP levels acquired before, during and after the induction protocol. Representative data demonstrating development of the DCM phenotype are shown in Figure 1.

No animals exhibited clinical signs of left sided congestive heart failure (pulmonary edema) during development of the DCM phenotype. One dog developed right sided congestive heart failure (ascites) which resolved with 2 days of reduced tachypacing rate. All dogs developed dilation of the LA and left ventricle (LV) as well as pronounced impairment in indices of systolic and diastolic function as illustrated by Figure 1. At enrollment the average LV ejection fraction was 34% ± 4, with a LV internal diameter in diastole (LVIDd) of 3.9 cm ± 0.1. Central jets of mitral regurgitation were present in 6/7 dogs due to annular dilation at enrollment (no disruption of the mitral apparatus was utilized for this study).

### Study Design

For the study days, all dogs had a baseline echocardiogram performed under butorphanol sedation (0.2–0.4 mg/kg IV) and a blood draw for analysis of NT-proBNP level (IDEXX Laboratories). Dogs were randomized to receive placebo (an empty gel capsule) or pimobendan. Pimobendan was dosed at 0.25 mg/kg (0.21–0.28 mg/kg) orally (Vetmedin, Boehringer Ingelheim); all dogs received one 2.5 mg tablet except one dog (13.9 kg), who received 1.5 tablets (3.75 mg). Three hours after their oral dose, the dogs were sedated and post-dose echocardiograms and a blood draw for NT-proBNP were performed. One week later, the procedure was repeated with dogs receiving the other treatment (placebo or pimobendan) to complete the crossover. The echocardiographer (KAJ) was blinded to the dosing until after all measurements were compiled. For these studies, cardiac pacing was discontinued (switching the pulse generator to ODO mode) ~30 min prior to each echocardiogram as previously described (20); pacing was resumed at the maintenance rate (180 ppm) during the 3 hour period after dosing.

### Echocardiography

Right-sided short axis parasternal images were used for M-mode measurements, 2D LA:Ao (using the Swedish method), fractional area change, and circumferential strain. Fractional area change was calculated from left ventricular area tracings excluding the papillary muscles in systole and diastole. Strain was calculated by lining up the QLab aCMQ (automated cardiac motion quantification, Philips Ultrasound) tracing with the 2D cine of the ventricle, the computer system tracked the movement through a cardiac cycle. Long axis right parasternal views were used for ejection fraction (EF) measurements *via* the Simpson's method of discs. Pulsed wave Doppler and TDI waves were recorded from left apical four and five-chamber views. Velocity time integrals (VTIs) were measured by tracing flow profiles and used in LV outflow volume calculations. Systolic time intervals were assessed from the left ventricular outflow tract (LVOT) trace paired with the simultaneous ECG. Doppler waves were measured according to standard practices (21) LA maximum and minimum volumes, jet areas, and continuous wave measurement of MR were taken from left apical four and two chamber (orthogonal) views. LA volumes were estimated based on these two views (“biplane method”) (22) using Simpson's method of discs. LA maximum (LA Vmax) and minimum sizes (LA Vmin) were used to calculate total LA emptying fraction (LA EF) and left atrial functional index (LAFI). Left atrial functional index is a value used clinically in humans (23) and is calculated with the following equation.

$$\begin{array}{l} \frac{LA\;EF\times LVOT\;VTI}{LA\;ESVI}\text{ where }LA\;ESVI=\frac{LA\;Vmax}{body\;surface\;area} \end{array}$$

MR fraction, EROA, and the ratio of ventricular stroke volume to aortic stroke volume were calculated according to the method described by Larouche-Lebel et al. (24). Mitral regurgitant jet area was measured by tracing the color jet (at a standardized Nyquist limit of −70 cm/s) in orthogonal planes and averaged, similar to the method described by Muzzi et al. (25). LA pressure can be approximated by E:e′ (26). IVRT was measured from the end of the aortic flow profile to the beginning of the mitral E wave flow profile. Studies were performed with a Philips CX50 system and S-8 ultrasound probe and all measurements were performed after study completion by a single operator, KAJ. All parameters were measured in triplicate and averaged. Studies were reviewed for accuracy by a board-certified cardiologist, KP.

### Statistical Methods

Two tailed paired *T*-tests were performed to evaluate mean differences between post-treatment placebo and pimobendan measurements, as well as to compare percent changes (from each respective pre-dosing baseline) with each treatment. Post-dose measurements were subtracted from that day's baseline measurement for each dog, then converted to a percent change. Data in tables are reported as the mean of the 7 dogs' values and the mean of the percent change from baseline. For this study, the significance threshold was set at *p* < 0.05, a priori. Correction was not performed for multiple comparisons, which is a limitation of the study. The normality assumption was tested with a Komolgrov-Smirnov normality test. The Wilcoxon Signed Rank Test was used for the MR area fraction because it did not pass the normality test. Pearson's correlation test was run to evaluate the pattern of change of the volume and area methods of MR quantitation.

This study utilized a colony of dogs that were induced into the DCM phenotype for preliminary studies of novel pharmacological agents. No power calculation was performed prior to this crossover study as a secondary aim was to evaluate the study methods for future studies with these animals.

## Results

### Systolic Function

**Figure 2:**
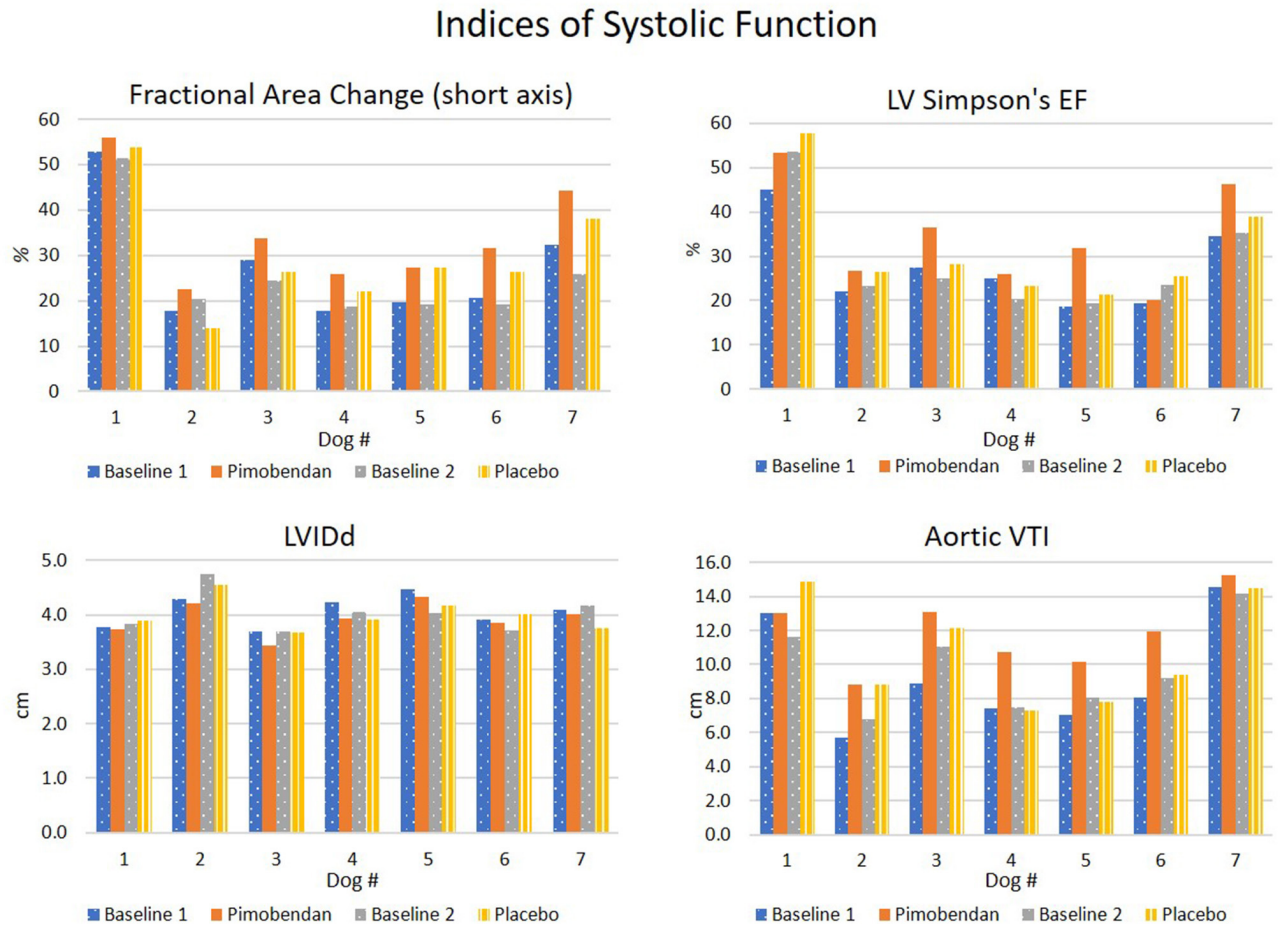
Summary of systolic function indices. LV Simpson's EF, LVIDd, left ventricular internal diameter in diastole from short axis M-mode; VTI, velocity time integral of aortic outflow.

As expected, pimobendan improved indices of systolic function, both when compared to placebo (Table 1) and baseline values (Figure 2).

**Table 1:** Measured echocardiographic variables.

	**Measured variables**	**Units**	**Baseline before placebo administration**	**Placebo**	**% change**	**Baseline before pimobendan administration**	**Pimobendan**	**% change**	***p*-value placebo vs. pimobendan**	***p*-value % change from baseline placebo vs. % change from baseline pimobendan**
Short axis M-mode	IVSd	cm	0.727	0.693	−3.9	0.761	0.729	−4.6	0.54	0.93
	LVIDd	cm	4.034	3.993	−0.8	4.066	3.930	−3.4	0.47	0.28
	LVPWd	cm	0.725	0.732	1.3	0.766	0.758	−0.7	0.34	0.66
	IVSs	cm	0.815	0.782	−2.5	0.814	0.857	5.3	**0.04**	0.35
	LVIDs	cm	3.355	3.256	−2.9	3.407	3.150	−7.3	0.07	0.06
	LVPWs	cm	0.951	0.920	−3.5	0.930	0.963	3.8	0.09	0.08
	HR	bpm	114	111	−2.7	122	97	−18.4	**0.008**	**0.05**
Swedish method	LA	cm	3.0	3.0	−1.2	3.1	3.0	−3.5	0.94	0.20
	Ao	cm	1.5	1.5	0.4	1.5	1.5	0.2	0.82	0.92
Short axis	LVAs	cm^2^	8.2	8.3	0.1	8.9	7.5	−15.0	**0.02**	**0.003**
	LVAd	cm^2^	11.1	11.7	6.1	12.0	11.4	−5.7	0.19	**0.02**
	Circumferential strain	(–)%	12.4	14.2	23.3	11.6	14.8	32.0	0.60	0.10
Long axis	LVs Vol	mls	34.8	33.9	−2.6	35.3	32.6	−8.0	0.49	0.25
	LVd Vol	mls	48.5	49.3	2.1	48.2	49.1	1.9	0.92	0.95
Left apical views	E	cm/sec	81.1	88.3	12.6	77.1	93.4	27.9	0.36	0.12
	A	cm/sec	42.9	43.5	9.6	44.5	49.5	12.3	0.36	0.91
	IVRT	sec	0.1	0.1	7.2	0.1	0.1	−1.9	0.68	0.39
	AVMax	cm/sec	80.5	88.3	8.8	77.0	94.2	24.5	0.24	0.08
	Aortic diameter	cm	1.2	1.2	0.2	1.2	1.2	0.3	0.61	0.98
	LVOT VTI	cm	9.8	10.7	9.5	9.2	11.9	34.9	0.13	0.06
	Mitral downslope	cm/s^2^	931.43	1005.17	45	973.36	1085.78	52	0.33	0.97
	PEP	sec	0.08	0.07	−10	0.07	0.07	−9	0.99	0.92
	AT	sec	0.07	0.07	6	0.06	0.07	7	0.38	0.92
	DT	sec	0.11	0.11	7	0.10	0.11	13	0.66	0.41
	LVET	sec	0.17	0.18	6	0.16	0.18	10	0.40	0.44
	MR VTI	cm	92.0	99.0	7.6	91.5	91.7	0.3	**0.02**	0.07
4 chamber left apical view	LA Min	mls	17.2	17.8	5.8	19.1	17.7	−7.3	0.77	**0.03**
	LA Max	mls	24.6	27.0	9.9	26.9	26.8	−0.5	0.77	**0.04**
	Regurgitant jet area	cm^2^	1.5	1.7	16.0	2.0	1.4	−40.3	0.07	**0.006**
	LA at same frame	cm^2^	8.7	9.3	7.9	9.8	9.5	−1.9	0.68	**0.009**
2 chamber left apical view	LA Min	mls	14.3	12.9	−8.5	15.3	13.4	−8.8	0.46	0.97
	LA Max	mls	20.9	20.2	−4.6	21.2	21.4	0.7	0.19	0.10
	Regurgitant jet area	cm^2^	1.2	1.4	−30.3	1.6	1.2	−14.2	0.52	0.51
	LA at same frame	cm^2^	8.7	8.6	−0.1	8.8	8.7	0.3	0.95	0.96
Medial	*e'*	cm/sec	8.1	8.7	7.1	9.0	8.7	3.5	0.74	0.34
	*a'*	cm/sec	6.9	6.0	−11.2	7.1	7.0	16.0	0.44	0.52
	*s'*	cm/sec	5.4	5.9	10.4	5.7	6.4	20.8	0.29	0.75
Lateral	*e'*	cm/sec	10.9	12.9	16.8	11.1	12.6	21.8	0.74	0.89
	*a'*	cm/sec	8.1	8.3	6.1	8.5	8.2	4.5	0.92	0.92
	*s'*	cm/sec	6.2	7.2	20.6	7.1	7.5	8.6	0.53	0.22

*% change refers to the average of each dog's value change from baseline to treatment. The view from which each measurement was taken is included on the left, with the timepoint and change in each variable in the column titles. Significant results appear in bold*.

Consistent with a direct inotropic action, pimobendan enhanced the fractional area change (FAC, *p* = 0.01) while decreasing the end-systolic LV area (LVAs) (*p* = 0.02) and increasing the systolic wall-thickness of the interventricular septum (IVSs, *p* = 0.04).

Heart rate (HR) (recorded from M-mode) was significantly decreased when dogs received pimobendan vs. when dogs received placebo (*p* = 0.008). Also, when given pimobendan, LV end-diastolic area (LVAd) percent changes from baseline decreased (*p* = 0.02), which would indicate a decrease in ventricular preload.

No ectopic beats or other arrhythmias were noted on the simultaneous ECG recording during echocardiographic exams. No adverse events (e.g., dyspnea, cough, vomiting) were seen in any of the dogs during the crossover study period.

### Left Atrial Function

Overall, when compared to placebo-treatment, pimobendan exerted negligible effects in the LA parameters LAFI, LA emptying volume, LA EF, LA:Ao, E:e′, and a wave velocity (see Table 1). However, when compared to pre-treatment values, both LA minimum and maximum volumes decreased following pimobendan administration while they increased in the placebo treatment data. This difference change was small but significant (4 chamber LA min −7% ± 3.8 pimobendan treatment, +6% ± 4 placebo treatment, *p* = 0.03) (4 chamber LA max −0.5% ± 3 pimobendan treatment, +10% ± 2 placebo treatment, *p* = 0.04) (LA max biplane −2% ± 3 pimobendan treatment, +3% ± 2 placebo treatment, *p* = 0.03).

### Mitral Regurgitant Volume Evaluation

**Figure 3:**
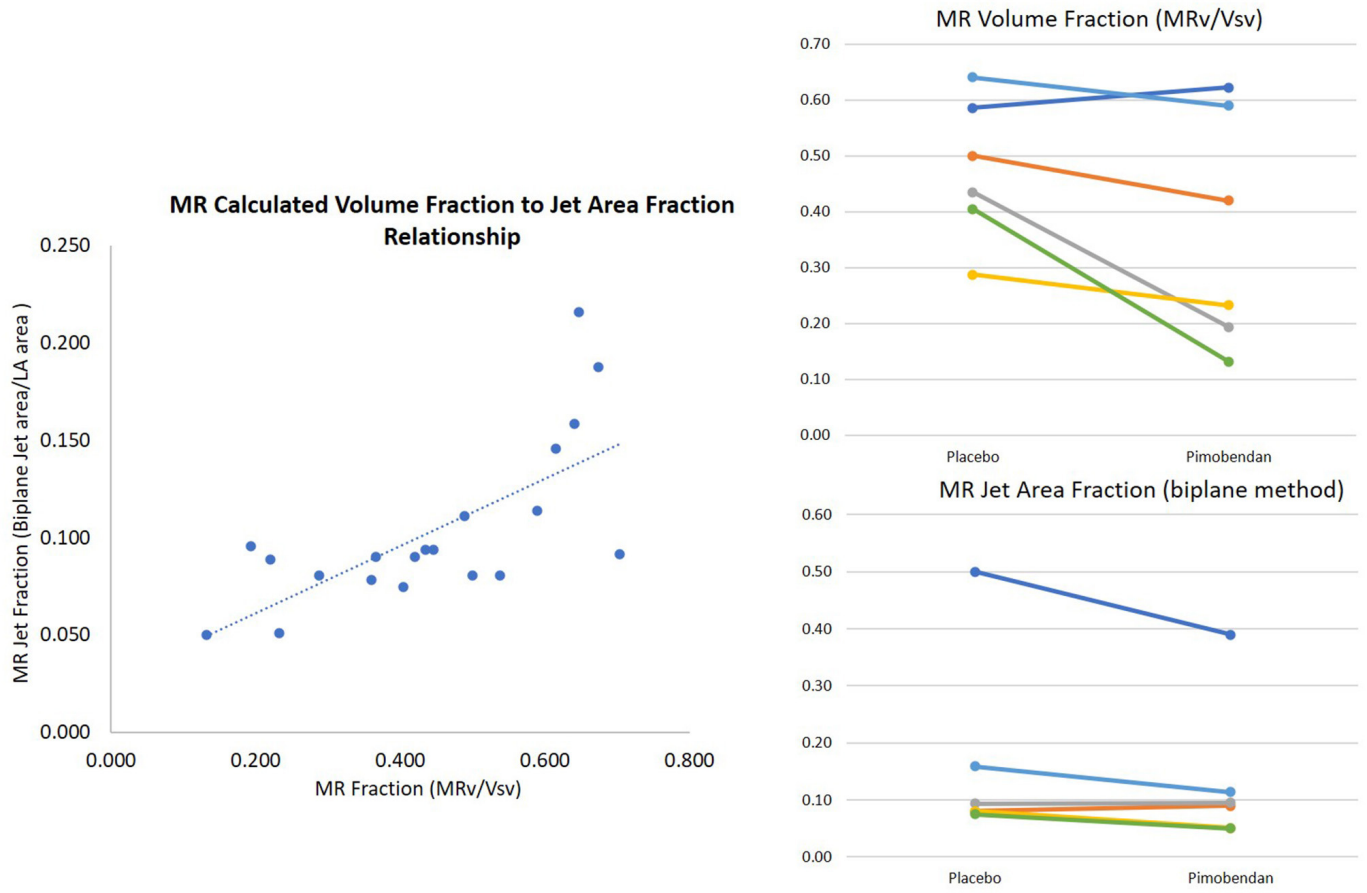
Mitral regurgitation quantification. Methods were positively correlated (*r* = 0.7 with high outlier removed) and found decreases in mitral regurgitation between placebo and pimobendan. Mitral regurgitant area fraction was significantly decreased with Wilcoxon Signed Rank Test *p* = 0.036. MR volume fraction was normally distributed with *p* = 0.07.

Overall, the two methods of MR fraction quantitation used in this study had a good correlation (r = 0.54 with *p* = 0.01) (Figure 3).

MR volume fraction was calculated according to the method described above (24). It was compared in dogs following the administration of pimobendan to values from the same dogs following the administration of placebo (pimobendan 0.36 ± 0.09, placebo 0.48 ± 0.05 *p* = 0.07). MR VTI was significantly lower in dogs following the administration of pimobendan with a mean of 92 cm ± 6 vs. a mean of 99 cm ± 7 (*p* = 0.02). The MR jet area fraction calculated as described (25) measured from the four-chamber view was also significantly lower following the administration of pimobendan compared to following placebo (*P* = 0.04) (Table 2).

**Table 2:** Calculated results from echocardiographic data.

	**Calculated variables**	**Units**	**Baseline before placebo administration**	**Placebo**	**% change**	**Baseline before pimobendan administration**	**Pimobendan**	**% change**	***p*-value placebo vs. pimobendan**	***p*-value % change from baseline placebo vs. % change from baseline pimobendan**
[(LVIDd-LVIDs)/LVIDd] × 100	FS	%	16.92	18.66	11	16.47	19.97	25	0.24	0.09
[(LVAd-LVAs)/LVAd] × 100	FAC	%	25.63	29.70	18	27.13	34.45	32	**0.007**	0.16
	E/A	ratio	1.91	2.12	14	1.72	1.93	17	0.50	0.87
	LA/Ao	ratio	1.96	1.93	−2	2.02	1.95	−3	0.65	0.57
(LA Min 2 chamber + LA Min 4 chamber)/2	Biplane Vmin	mls	15.73	15.37	−2	17.17	15.25	−10	0.75	0.16
(LA Max 2 chamber + LA Max 4 chamber)/2	Biplane Vmax	mls	22.78	23.56	3	24.08	23.65	−2	0.78	**0.03**
Biplane Vmax-Biplane Vmin	LA total emptying vol	mls	7.05	8.19	17	6.91	8.40	26	0.59	0.47
(Biplane Vmax-Biplane Vmin) Biplane Vmax	LA total emptying fraction		0.32	0.36	14	0.31	0.37	28	0.37	0.34
(LA EF × LVOT VTI)/ LA ESVI	LA FI		0.09	0.10	22	0.08	0.11	80	0.12	0.09
[(LVd Vol-LVs Vol)/LVd Vol] × 100	Simpson EF	%	28.59	31.61	11	27.44	34.42	27	0.28	0.13
LVIDd^3^-LVIDs^3^	Ventricular SV	mls	27.68	28.38	7	26.71	29.05	8	0.82	0.92
3.14159 × (Ao diam/2)^2^ × LVOT VTI	Aortic SV	mls	14.79	15.94	9	13.54	17.67	39	0.21	0.06
Ventricular SV-Aortic SV	MR volume	mls	15.51	12.43	21	14.69	11.68	−30	0.16	0.22
MR Vol/Ventricular SV	MR fraction		0.50	0.40	5	0.54	0.36	−36	0.07	0.15
4 chamber	Jet area/LA area		0.16	0.18	8	0.19	0.14	−40	0.036^*^	^**^
2 chamber	Jet area/LA area		0.14	0.15	41	0.17	0.13	−18	0.833^*^	^**^
(4 chamber + 2 chamber)/2	Biplane jet area/LA area		0.16	0.16	−4	0.18	0.13	−29	0.14^*^	^**^
MR Vol/MR VTI	EROA	cm^2^	0.19	0.16	11	0.17	0.14	−24	0.55	0.46
	IVRT/HR	ratio	0.000153	0.000159	4	0.000170	0.000134	−20	**0.05**	0.11
(4 chamber jet area + 2 chamber jet area)/2	LA regurgitant jet biplane	cm^2^	1.38	1.59	4	1.79	1.29	−29	0.059^*^	^**^
LA pressure estimate	E:e'	ratio	7.69	7.07	−2	7.08	7.85	17	0.09	0.28
	AT/LVET	ratio	0.38	0.38	0	0.40	0.38	−3	0.84	0.49
	PEP/LVET	ratio	0.45	0.37	−16	0.47	0.38	−18	0.82	0.82

*The equation used to acquire each variable is included on the left, with significant results appear in bold.*

* *Wilcoxon Signed rank test used instead of T-test.*

** *Evaluation not performed*.

### Other Indices

**Figure 4:**
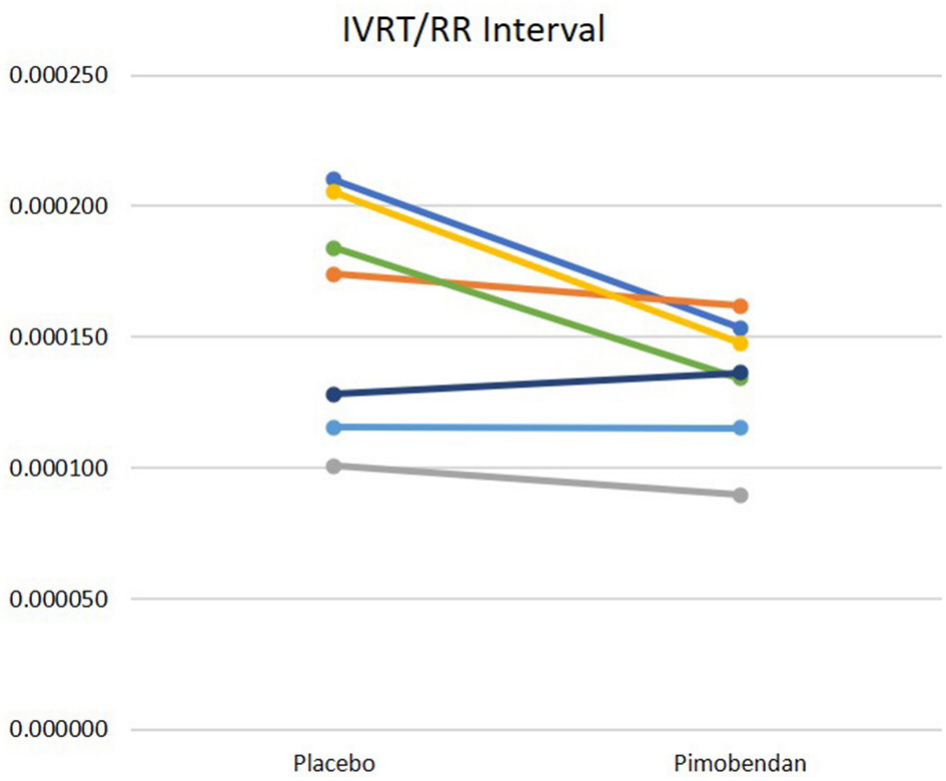
IVRT divided by RR interval. Data acquired after pimobendan was significantly different than data acquired after placebo, *p* = 0.05.

Pimobendan treatment shortened the IVRT when corrected for HR (Figure 4). However, other indices of diastolic function were not significantly different, including the E wave velocity and downslope, as well as the early mitral annular displacement velocity (e′). Similarly, no differences in aortic acceleration time (AT), pre-ejection period (PEP), LV ejection time (LVET), and AT/LVET, or PEP/LVET ratios were noted between the pimobendan and placebo data sets; no rate-corrections were studied for these intervals.

### NT-proBNP Concentration

**Figure 5:**
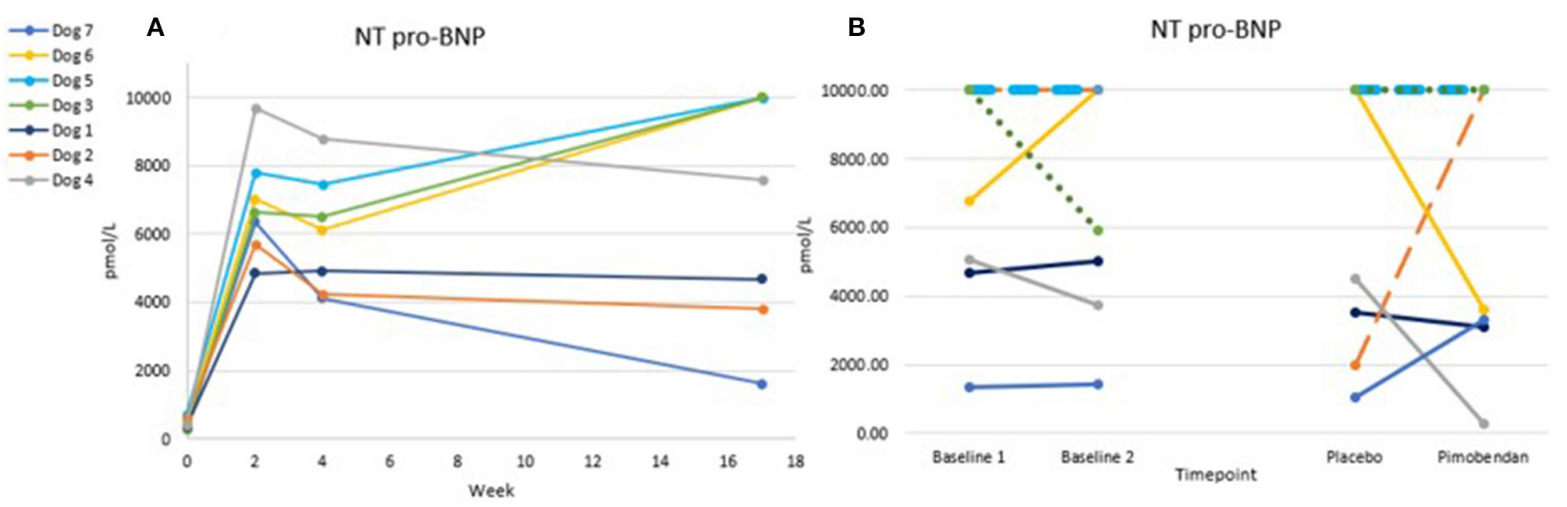
Chart **(A)** shows trends of NT pro-BNP over time from baseline through tachypacing. Chart **(B)** shows the variability in this value from Baseline 1 to Baseline 2 (1 week apart) and the timepoint 3 h after oral dosing. Lines are dashed due to overlap. No significant difference was found between pimobendan and placebo *p* = 0.95.

The tachypacing protocol induced marked elevations in NT-pro BNP values (Figure 5), in line with the induced DCM phenotype. Only 4/24 proBNP values were below the 1,800 pmol/L threshold provided by the reference lab for clinically significant heart disease, and all but one value were over the 900 pmol/L upper limit of the normal range. NT-proBNP level was not significantly different when dogs were administered placebo vs. when they were administered pimobendan, though higher variability was seen in the post-dosing results compared to the baseline results.

## Discussion

Indices of systolic function showed significant improvements with pimobendan administration including FAC and IVSs, however Simpson's EF and fractional shortening were not found to change. Surprisingly, the post-placebo data also had positive percent changes in systolic parameters such as Simpson's EF, FAC, and fractional shortening compared to baseline (Table 2). Whether these are due to stress (second echo of the day), time of day (morning vs. midday), or the break from tachypacing provided by the first echo, are unknown, and they were not tested for statistical significance. Despite this pattern, comparisons to the data acquired when animals were given placebo still provided the opportunity for assessment of drug induced changes.

LV chamber dimensions (LVAs and LVAd) decreased in both systole and diastole, consistent with a previous study (16). However, left ventricular strain, a parameter considered to be a surrogate for contractility did not change significantly in our study. A study in humans that found significant improvements in echocardiographic strain indices with levosimendan used complex methods to reduce the dilator function of the medication. The methods involved manipulating pressures with phenylephrine-induced vasoconstriction (27). The decreases in LV area and overall size induced by pimobendan decrease wall tension, which in turn decreases the cardiac strain. While the inotropic effect should increase the strain value, we propose the measurable end result is mixed due to the vasodilator function of this medication.

Atrial function can be difficult to evaluate independently from ventricular function, as the ventricular diastolic function determining the “sucking” effect during filling has a direct effect on the maximum and, to a lesser degree, on the minimum size of the atria. Therefore, the calculated atrial emptying fraction includes a passive component, and is not solely due to atrial myocyte contraction. However, in states where atrial function is severely compromised and atrial contraction is lost, such as atrial fibrillation, negative clinical effects are apparent, highlighting the importance of understanding pharmacological effects on these chambers independent of the ventricles. In an attempt to measure atrial inotropy, a number of echocardiographic parameters were analyzed. The echocardiographic parameters of atrial function included LA minimum and maximum size with biplane method, LA EF, LAFI, mitral A wave velocity, and medial and lateral a′ wave velocity. None of these echocardiographic parameters showed obvious differences compared to placebo. However, there were significant decreases in LA minimum and maximum size after pimobendan administration when compared to baseline but increases in these values after placebo administration compared to baseline in the 4 chamber view but not the 2 chamber view. Reductions in LA minimal volumes can be associated with direct atrial inotropism (28). This finding may support a positive inotropic effect of pimobendan on the atria, but we did not find strong evidence to support this hypothesis. This change may alternatively be explained by improvements in ventricular lusitropic function, as our results did show evidence of this effect. The recent study by Sarcinella et al. (9) of DMVD dogs administered pimobendan chronically which found no significant improvements in LA function may be more characteristic of its effect in dogs.

Pulmonary vein flow and auricular flow could have given further insights into LA function but were not assessed during this study and are not routinely obtained echocardiographic parameters in dogs with DCM. Other studies have shown decreased LA pressure by direct measurement in dogs with experimentally induced MR given pimobendan (29), so though we would have expected to find this change with our LA pressure estimate E:e′, a change was not captured by our methods.

Quantification of MR *via* echocardiography can be challenging and each method has inherent biases and sources of error. We used a color jet-based method along with a volume calculation method that have been previously described, in addition to tracing the continuous-wave Doppler signal of the MR for VTI. Two out of three measures (volume, area, and VTI) used to quantify MR decreased significantly when dogs were treated compared to when given placebo. These findings, in the face of the above-mentioned increases in systolic function, refute the theory that increased inotropy necessarily increases regurgitant volume. While this has been suspected in DMVD (10), in the presence of functional mitral valve regurgitation as seen in DCM, pimobendan appears to decrease the volume of MR. As previously discussed (30) we hypothesize that the decrease in LV chamber size allows the mitral leaflets to coapt more effectively leading to the decrease in MR volume. This may be true even with an insufficient valve as in DMVD (2) though this is inferred from heart size rather than direct MR measurement. Our findings support the theory that oral pimobendan administration exerts an additional benefit from positive inotropy, which is a decrease in mitral regurgitant volume in dogs with a DCM phenotype.

Our canine model of DCM showed notable impairment of lusitropy after the tachypacing protocol as compared to pre-pacing. This was illustrated by lengthening of the IVRT (Figure 1) and decreases in e′ velocities (15.1 cm/s pre-pacing to 10.7 cm/s post-pacing). Improvements in cardiac relaxation attributable to pimobendan administration were documented in IVRT normalized to HR (Figure 4). However, pimobendan did not have a statistically significant effect on e′ velocities compared to placebo (Table 1). An explanation for this result may be our small sample size, as in one dog, we were unable to evaluate this parameter due to suboptimal TDI quality. The indicator of improved lusitropic function that echocardiography was able to capture in this study was IVRT/HR, an effect consistent with the findings of previous studies (14, 15). This finding, in combination with the significantly lower HR when dogs were treated (*p* = 0.01) also seen in other studies (30) may have a considerable contribution to the increase in longevity seen with pimobendan treatment.

The high variability in the post-dosing levels of NT-proBNP may be due to various factors including sedation, stress, volume status, and changes in HR. We therefore do not recommend using this value in this study design, though it may be amenable to chronic dosing studies or those with longer post-dose timepoints. Possible explanations for 4/24 samples not exceeding the 1,800 pmol/L threshold may be exhaustion of the hormonal signaling system responsible for this molecule. Studies recommend a lower threshold value for dogs with DCM rather than DMVD due to this phenomenon (31, 32). Also, generally this test is used for screening for evidence of heart disease, its negative predictive value is not widely emphasized.

Limitations of our study include the fact that only one dose of medication was administered and the dose was fixed. Myocytes from tachypacing hearts show decreased response to inotropes in general (33) which may be why more dramatic changes in all systolic function parameters were not found. The FDA labeled dose for pimobendan was administered for this study because this is the most common dose used in clinical medicine and proven to be safe and effective. Additionally, oral pimobendan dosing is used in hospitalized patients with active congestive heart failure, thus understanding how a single dose may benefit our patient is clinically relevant and may impact treatment decisions. However, it was not investigated whether administering increased dosages would have produced more profound effects.

Other limitations included the small sample size of our study, with only 7 dogs available to be evaluated. This, along with the many parameters evaluated and a lack of correction for multiple comparisons lead to a concern for type 1 error. Our goals of thorough echocardiographic evaluation of LA function and MR specifically led us to perform so many different measurements, as no one measurement is without limitations. This wide net of parameters unfortunately drastically increases the chance that our significant findings include statistical error. However, no statistically significant findings occurred that opposed the hypotheses of this study, and the significant changes we found are all consistent with the literature and previously characterized effects of pimobendan.

The tachypacing model of heart failure shows many similarities structurally and functionally to naturally occurring DCM (34). It is also similar to a cardiac phenotype caused or exacerbated by pathologic tachycardias such as atrial fibrillation or other supraventricular tachycardias. Therefore, we believe our findings should be relevant to clinical veterinary patients. Markedly dilated chambers, thinned walls, reduced ejection fraction, and chamber dyssynchrony are all factors that these hearts have in common.

Despite our best efforts to control for breed, age and tachypacing treatment, there was variability in the heart failure exhibited by our study dogs. One dog had significantly worse MR than the others and also exhibited a higher EF (49% compared to average of 28%). One dog had only scant MR thus we were unable to accurately obtain quantification parameters. However, the variability in degree of MR in our study population likely reflects the variability seen in clinical patients that present with a DCM phenotype thus we elected to include these dogs' data in our analyses. Lastly, some differences exist between the tachypacing model of heart failure and intrinsic DCM. One example is the lack of arrhythmia present in our study population, whereas some degree of ventricular ectopy or supraventricular tachycardia is extremely common in dogs affected by naturally occurring DCM.

In conclusion, the results of our study are supportive of the hypothesis that pimobendan decreases MR and shortens IVRT in dogs with an experimentally induced DCM phenotype. Evidence of improved systolic function was present as well as a clear decrease in HR. LA size decreased compared to baseline when dogs were given pimobendan but other measures of LA function were not seen to change, and no significant change in NT-proBNP concentration was demonstrated.

## Data Availability Statement

The raw data supporting the conclusions of this article will be made available by the authors, without undue reservation.

## Ethics Statement

The animal study was reviewed and approved by the Institutional Animal Care and Use Committee (IACUC) of QTest Labs.

## Author Contributions

KA-J, SR, CR, and RH conceived study design and methodology. KA-J carried out experiments, compiled data, performed statistical analyses, and prepared the manuscript. KP, CR, and RH reviewed and edited the manuscript. All authors contributed to the article and approved the submitted version.

## Conflict of Interest

CR was employed by the company MyoKardia, Inc. The remaining authors declare that the research was conducted in the absence of any commercial or financial relationships that could be construed as a potential conflict of interest.

## Abbreviations

DCM: Dilated cardiomyopathy
HR: Heart rate
DMVD: Degenerative mitral valve disease
MR: Mitral regurgitation
LA: Left atrium
LV: Left ventricle
EROA: Effective regurgitant orifice area
TDI: Tissue Doppler imaging
IVRT: Isovolumetric relaxation time
FAC: Fractional area change
EF: Ejection fraction
LA EF: Left atrial emptying fraction
LAFI: Left atrial functional index
LVID: Left ventricle internal diameter
IVS: Interventricular septum
VTI: Velocity time integral.
